# The Disease Burden of Primary Intracerebral Hemorrhage in Hunan Province, China in 2018

**DOI:** 10.1007/s44197-022-00045-5

**Published:** 2022-05-28

**Authors:** Linzhi Han, Tao Tang, Ying Lin, Jingcheng Shi, Hua Zhong, Mengshi Chen, Jing Deng, Wei Zhang

**Affiliations:** 1 Department of Clinical Pharmacology, Xiangya Hospital, Central South University, 87 Xiangya Road, Changsha, 410008 China; 2 Tongxiang Center for Disease Control and Prevention, Zhejiang, 314500 China; 3 Hunan Provincial Key Laboratory of Clinical Epidemiology, Xiangya School of Public Health, Central South University, Changsha, 410008 China; 4 Department of Cardiology, Xiangya Hospital, Central South University, Changsha, 410008 China; 5 Institute of Clinical Pharmacology, Central South University, Hunan Key Laboratory of Pharmacogenetics, Changsha, 410008 China; 6 Engineering Research Center of Applied Technology of Pharmacogenomics, Ministry of Education, 110 Xiangya Road, Changsha, 410008 China; 7 National Clinical Research Center for Geriatric Disorders, Xiangya Hospital, Central South University, Changsha, 410008 China

**Keywords:** Primary intracerebral hemorrhage, Disease burden, Disability-adjusted life years

## Abstract

**Background:**

Hunan Province is a region in China with a high prevalence of intracerebral hemorrhage, especially primary intracerebral hemorrhage (PICH). The objective of this observational study was to assess the disease burden of PICH.

**Methods:**

We searched the Hunan Provincial Health Statistics Direct Reporting and Decision Analysis System to retrieve PICH inpatient and outpatient data and all-population all-cause deaths in Hunan Province in 2018. DisMod II was used to estimate the disability-adjusted life years (DALYs) due to PICH in 2018.

**Results:**

In 2018, 30,400 new PICH cases were recorded in Hunan Province. The incidence was higher among men (51.6 per 100,000) than women (29.3 per 100,000). The DALYs due to PICH were 478,000 patient-years, the years of life lost (YLLs) were 452,000 patient-years and the years lived with disability (YLDs) were 27,000 patient-years. In 2018, the rate of DALYs due to PICH was 6.4 patient-years per 1000 individuals, the rate of YLLs was 6.1 patient-years per 1000 individuals, and the rate of YLDs was 0.3 patient-years per 1000 individuals.

**Conclusion:**

We estimated the DALYs due to PICH in Hunan Province in 2018, thereby providing relevant data for the development of policies and measures for the management of PICH disease burden.

**Supplementary Information:**

The online version contains supplementary material available at 10.1007/s44197-022-00045-5.

## Introduction

Primary intracerebral hemorrhage (PICH) is defined as intraparenchymal hemorrhage unrelated to trauma or vascular structural lesions. PICH is the most common type of intracerebral hemorrhage (ICH), with a high mortality rate. It is estimated that the global incidence is 18 per 100,000 individuals. In Asian populations, the incidence is 21 per 100,000 individuals. The 30-day mortality rate is 25%, and the 3-month mortality rate is 42.5% [1–4]. In 2017, PICH accounted for 26.2% of 11.931 million stroke events worldwide.

Several epidemiological surveys of the prevention and treatment of cerebrovascular diseases in China from 1980s showed that the incidence and prevalence of ICH in Hunan Province, especially in the city of Changsha, were higher than those in other regions of the country [5–8]. The annual incidence of ICH was 131.1 per 100,000 individuals and the adjusted annual incidence for ICH was 73.1 per 100,000 individuals from 1986 to 2000 after adjusted by the world population in 1985 for age and sex [9].Surveillance of stroke incidence from three cities in China during the 1990s also showed that the overall age-adjusted incidence for ICH was highest in Changsha 77.1/100,000), followed by Beijing (38.1/100,000) and Shanghai (23.1/100,000), as well as in both the male and female populations [6]. In comparison, the global incidence of ICH was 82 per 100,000 individuals in 2010 [8]. In China, the overall incidence of ICH was 82.1 per 100,000 individuals in 2013 and the age-adjusted incidence for males (87.2) was higher than females (77.1) [7]. Hunan is a province with medium economic development in central and southern China. According to the results of the seventh national census, the number of people over the age of 60 in Hunan Province is 19.88%, an increase of 5.34% over 10 years ago. The degree of aging ranks 10th in China, higher than the national average. With the disparity in the aging process, the region variations in the incidence of ICH across China will continuously exist.

The ICD-10 code does not code PICH alone; PICH is included in intracerebral hemorrhage I61, and the often-said hemorrhagic stroke also includes subarachnoid hemorrhage. Therefore, the current disease burden statistics based on ICD codes often mix the disease burden caused by PICH with that caused by the total stroke or hemorrhagic stroke. For example, the estimated DALYs of Global Burden of Disease (GBD) global hemorrhagic stroke patients in 2013 (65.45 million person-years) [10] and the estimated disability-adjusted life years (DALYs) and age-standardized DALY rates of hemorrhagic stroke in China in 2016 (22.408 million person-years and 1427.3 person-years, respectively) [11] include I60, PICH, and secondary ICH. Due to the different causes of ICH, the corresponding treatment and prevention measures also differ. The main cause of PICH is hypertension. With the prevention of hypertension in high-incidence areas, such as Hunan, what is the burden caused by PICH? An analysis of the burden of disease caused by PICH can provide a reference for health administration aiming to further develop targeted measures against hypertension.

In this study, we collected PICH outpatient and inpatient data, the demographics of the resident population, and all-population all-cause deaths in Hunan Province in 2018 to estimate the DALYs due to PICH using DisMod II, a GBD analysis template.

## Materials and Methods

### Data Source

We obtained the PICH inpatient and outpatient data and all-population all-cause deaths in Hunan Province from the Hunan Provincial Health Statistics Direct Reporting and Decision Analysis System established by the Hunan Provincial Health Commission. The system contains inpatient and outpatient data from level 2 and above public hospitals in Hunan Province. According to China’s “Hospital Classification Management Standards”, secondary Level hospitals provide comprehensive medical and health services to multiple communities. Tertiary level hospitals provide high-level medical and health services to the region and surrounding radiation areas. Cardiovascular and cerebrovascular diseases, such as stroke, heart attack and other diseases, are generally transferred to secondary and above hospitals for treatment. Moreover, we obtained the demographic information of the resident population in Hunan Province from the Hunan Statistical Yearbook 2019 [12].

The Hunan Provincial Health Statistics Direct Reporting and Decision Analysis System was established by the Hunan Provincial Health Commission in 2012. The system includes the demographics of Hunan Province, the medical records of outpatients and inpatients at level 2 and above public hospitals in Hunan Province, basic data of health resources, basic public health services and other data, and these data can be exported as needed for further analysis to maximize the value of health statistics. Moreover, the system provides accurate, convenient access to health authorities, thereby providing information support and a scientific basis for developing health policies and decision making. Hunan Statistical Yearbook 2019 is prepared by the Hunan Provincial Bureau of Statistics, which systematically includes a large number of statistics on the economic and social development of Hunan Province in 2018 in cities, continents and counties (cities and districts), divided into 22 chapters, and is an informative annual publication that comprehensively reflects the economic and social development of Hunan Province.

### Extraction and Exclusion of PICH Data

The data were exported from the medical records of inpatient and outpatient data in the Hunan Provincial Health Statistics Direct Reporting and Decision Analysis System. This system contains data related to inpatient and outpatient care in public hospitals above the second level in Hunan Province. With the national wide development of The Chinese Stroke Center Alliance (CSCA), the Stroke first aid green channel in the emergency department is common established and well run in every prefecture-level city. Cardiovascular and cerebrovascular diseases such as stroke, heart attack, and other diseases are generally transferred to secondary and above hospitals for treatment. The original database includes the following variables: name, sex, age, ID, date of admission, diagnosis, and ICD-10 code. Each record in the database was screened for inclusion or exclusion based on the following criteria.

#### Data Extraction

1. Date of admission or clinic visit: January 1, 2018 to December 31, 2018;
2. Inpatient or outpatient records in which ICH was mentioned in the diagnosis or I61 was included in the diagnosis code (ICD-10); and
3. All records with a diagnosis of I61 were reviewed to confirm that the diagnosis was based on brain computed tomography (CT)/magnetic resonance imaging (MRI) or surgical diagnosis within 2 weeks of onset.

#### Data Exclusion

1. ICH with subarachnoid hemorrhage (ICD-10 code I60);
2. ICH with cerebral infarction (ICD-10 code I61 with CT/MRI-confirmed postinfarction hemorrhage);
3. ICH due to trauma or vascular structural lesions (with the exact trauma cause, CT/MRI-confirmed vascular malformations);
4. Nonacute ICH (28 days or more since onset); and

The ID was used as the unique identification, and the name was used for verification. Patients with recurrent conditions were counted as one case if their condition reoccurred within 28 days or multiple cases if their condition recurred after 28 days. New cases of PICH patients were defined as the first admission or visit to the clinic due to ICH in 2018.

### Statistical Analysis

The incidence, remission rate, and mortality rate of PICH per age and sex were input into DisMod II software to compute all indicators required to calculate the DALYs. The incidence of PICH in Hunan Province in 2018 was calculated by the number of new PICH cases per age and sex divided by the resident population in Hunan Province in 2018. The remission rate differs by age and sex; according to Wei C’s study, the remission rate is 0 during the chronic phase. The mortality rate within 28 days is set at 36.3%, and the mortality rate after 28 days is set at 69.2% [13, 14]. Then, all indicators were imported into the DALY template from the World Health Organization (WHO) to calculate the years of life lost (YLLs), the years lived with disability (YLDs), and the DALYs. The DALYs were calculated using DisMod II software and the WHO’s DALY template. The rates and their 95% confidence intervals (95% CIs) were analyzed with SPSS v21.0.

## Results

### The Incidence of PICH in Hunan Province in 2018

In 2018, 30,400 new PICH cases were recorded in Hunan Province, with an overall incidence of 40.8 per 100,000 individuals. The incidence among men (51.6 per 100,000 individuals) was higher than that among women (29.3 per 100,000 individuals) (= 2263.0, p < 0.001). The incidence of PICH increased with age, with a rapid increase among those aged 40 years or older and a peak among those aged 70–80 years (Table 1).

**Table 1:** The incidence of PICH and PICH cases per age and sex in Hunan Province in 2018 (per 100,000 individuals, *n*, 95% CI)

Age (years)	Men		Women		Total	
	*n*	Incidence	*n*	Incidence	*n*	Incidence
0 ~	29	0.6 (0.4–0.8)	18	0.4 (0.2–0.6)	47	0.5 (0.3–0.6)
10 ~	248	5.5 (4.8–6.2)	140	3.7 (3.1–4.4)	388	4.7 (4.2–5.2)
20 ~	488	10.1 (9.2–11)	235	5.1 (4.5–5.8)	723	7.7 (7.1–8.2)
30 ~	1339	22.4 (21.2–23.6)	432	7.5 (6.8–8.2)	1771	15.1 (14.4–15.8)
40 ~	2946	47.9 (46.2–49.7)	1259	22.2 (20.9–23.4)	4205	35.6 (34.5–36.6)
50 ~	4592	84.3 (81.8–86.7)	2565	48.8 (46.9–50.7)	7157	66.8 (65.3–68.4)
60 ~	5090	133.7 (130.1–137.4)	2762	75.9 (73–78.7)	7852	105.4 (103.1–107.8)
70 ~	3679	201.1 (194.6–207.6)	2146	118.1 (113.1–123.1)	5825	159.7 (155.6–163.8)
80 ~	1423	176.4 (167.3–185.6)	1009	101.4 (95.1–107.6)	2432	135 (129.6–140.3)
Total	19,834	51.6 (50.9–52.3)	10,566	29.3 (28.7–29.9)	30,400	40.8 (40.3–41.3)

The incidence among men (51.6 per 100,000 individuals) was higher than that among women (29.3 per 100,000 individuals) (*χ*^2^ = 2263.0, *p* < 0.001)

### YLLs and YLDs due to PICH in Hunan Province in 2018

In 2018, the YLLs due to PICH were 452,000 patient-years (men: 292,000 patient-years, 64.7%; women: 160,000 patient-years, 35.3%). The rate of YLLs was 6.1 patient-years per 1000 individuals (men: 7.6 patient-years per 1000 individuals; women: 4.4 patient-years per 1000 individuals). The YLD was 27,000 patient-years (men: 18,000 patient-years, 65.2%; women: 9000 patient-years, 34.8%). The rate of YLD was 0.2 patient-years per 1000 individuals (men: 0.5 patient-years per 1000 individuals; women: 0.3 patient-years per 1000 individuals) (Supplemental Table 1).

### DALYs due to PICH in Hunan Province in 2018

In 2018, the DALYs due to PICH were 478,000 patient-years (YLLs: 94.4%, YLDs: 5.6%; men: 64.7%, women: 35.3%). The rate of DALYs was 6.4 patient-years per 1000 individuals (men: 8.0 patient-years per 1000 individuals; women: 4.7 patient-years per 1000 individuals) (Table 2).

**Table 2:** DALYs due to PICH (patient-years, 95% CI) and the rate of DALYs (patient-years per 1000 individuals, 95% CI) in Hunan Province in 2018

Age (years)	Men		Women		Total	
	DALYs	Rate of DALYs	DALYs	Rate of DALYs	DALYs	Rate of DALYs
0 ~	319.4 (203.2–435.7)	0.1 (0.1–0.2)	201.2 (108.3–294.2)	0.1 (0.0–0.1)	520.7 (311.4–729.9)	0.1 (0.1–0.2)
5 ~	3387.9 (2830.1–3945.6)	0.7 (0.6–0.8)	1975.8 (1544.3–2407.3)	0.5 (0.4–0.6)	5363.7 (4374.4–6353)	0.6 (0.5–0.7)
15 ~	16,251.9 (14,657.8–17,845.9)	2.3 (2.1–2.6)	8362.1 (7200.9–9523.3)	1.3 (1.1–1.5)	24,613.9 (21,858.7–27,369.2)	1.9 (1.6–2.1)
30 ~	58,603.9 (55,786.9–61,420.8)	6.7 (6.4–7)	22,358.1 (20,610.9–24,105.2)	2.7 (2.5–2.9)	80,962 (76,397.8–85,526)	4.7 (4.5–5)
45 ~	117,704.3 (113,975.1–121,433.4)	13.3 (12.9–13.7)	63,784.3 (60,976.4–66,592.2)	7.6 (7.3–7.9)	181,488.6 (174,951.5–188,025.6)	10.5 (10.1–10.9)
60 ~	70,279.5 (68,339.2–72,219.8)	18.5 (18–19)	42,493.5 (40,903.1–44,084)	11.7 (11.2–12.1)	112,773 (109,242.2–116,303.8)	15.1 (14.7–15.6)
70 ~	34,447.6(33,358.6–35,536.7)	18.8 (18.2–19.4)	22,991.1 (22,034.2–23,947.9)	12.7 (12.1–13.2)	57,438.7 (55,392.8–59,484.6)	15.8 (15.2–16.3)
≥ 80	8492 (8090.2–8893.7)	10.5 (10–11)	6823 (6431.9–7214.1)	6.9 (6.5–7.2)	15,314.9 (14,522–16,107.9)	8.5 (8.1–8.9)
Total	309,486.4 (297,241.1–321,731.6)	8 (7.7–8.4)	168,989.1 (159,809.8–178,168.3)	4.7 (4.4–4.9)	478,475.5 (457,050.9–499,899.9)	6.4 (6.1–6.7)

## Discussion

This study shows that the incidence of PICH was 40.8 per 100,000 individuals in Hunan Province in 2018, which is higher than the global overall incidence (18 per 100,000 individuals) and the incidence in Asian populations (21 per 100,000 individuals) [4, 15]. The incidence of PICH increased with age and peaked at 70–80 years of age among both men and women, a finding consistent with chronic disease trends and the age distribution of ICH in Tianjin in 2015 [13]. The incidence of PICH decreased in the group over 80 years old, which is an illusion caused by cross-sectional data. Because the occurrence of PICH in the elderly is not only related to the increase of age, but also affected by their age of birth and growth exposure experience.

Hypertension is a major risk factor for PICH; therefore, the high incidence of PICH in Hunan Province may be related to the high prevalence of hypertension, which was 44.71% in adults in 2015, while the global prevalence of hypertension was 31.1% in adults in 2010, and the prevalence of hypertension in East Asia, South Asia, and Southeast Asia was 31.5% [16, 17]. The incidence of PICH increased with age and peaked at 70–80 years of age among both men and women, a finding consistent with chronic disease trends and the age distribution of ICH in Tianjin in 2015 [13]. This study shows that the incidence of PICH among men (51.6 per 100,000 individuals) was higher than that among women (29.3 per 100,000 individuals) in Hunan Province. This finding was similar to a previous study, which showed that the incidence of ICH among men was also higher than that among women in Changsha [6]. Although this result differs from the findings of a meta-analysis [18] that showed no sex-related variation in the incidence of PICH. However, we believe that given the sex-related variation in hypertension and lifestyle, two major risk factors for PICH, it is natural to find sex-related variation in the incidence of PICH in Hunan Province. Xie et al. [16] reported that the overall incidence of hypertension among men (51.6/100,000) was higher than that among women (29.3/100,000) among residents ($\ge$ 18 years old) of Hunan Province from 2007 to 2015. Similarly, Wang et al. [17] also reported that the estimated global age-standardized prevalence of hypertension in adults aged $\ge$ 20 years in 2010 was 31.1% (95% CI 30.0–32.2%): 31.9% (30.3–33.5%) in men and 30.1% (28.5–31.6%) in women. Moreover, in terms of lifestyle, men are more likely than women to form bad habits such as smoking, drinking, and low intake of vegetables and fruits, thereby increasing the risk of PICH [19].

In 2018, in Hunan Province, the YLL due to PICH was 452,000 patient-years, and the YLD was 27,000 patient-years. Both the YLLs and YLDs were higher among men than women across age groups. Among both men and women, the YLLs and YLDs peaked at 45 years of age. The rate of YLLs and the rate of YLDs peaked later than those of YLLs and YLDs due to variation in the resident population numbers across the age groups. In 2012, the rate of YLLs due to stroke, including hemorrhagic stroke and ischemic stroke, was 3.61 patient-years per 1000 individuals in Shanghai [20]. In our study, the rate of YLLs due to PICH alone reached 6.1 patient-years per 1000 individuals, indicating that the burden of PICH in Hunan Province is more prevalent and severe than in Shanghai [20]. One possible reason might be the economic level of Shanghai was higher than that of Hunan. As we know, socioeconomic status plays an important role in health disparity, which affects the accessibility of medical and health resources. A Systematic Analysis of the Global Burden of Disease Study 2017 showed a trend toward the greater burden of ICH in high-income countries and slightly reduced burden of ICH in low- to middle-income countries, which Mongolia, Turkmenistan, and Papua New Guinea demonstrated the highest incidence, mortality, prevalence, and DALY rates of ICH, while Switzerland, New Zealand, and Australia showed the lowest rates [21].

Our study shows that the DALYs due to PICH in Hunan Province were 478,000 patient-years in 2018. Another study used DISMOD-MR software to calculating DALYs and showed that the DALYs due to ICH in Hunan Province were 1.2212 million patient-years in 2017 [22]. Despite the two studies are slightly different in estimating the incidence and mortality rate, we can still can deduce with cautions that DALY caused by PICH accounts for about approximately 39.1% of all DALYs due to ICH. YLLs accounted for 94.4% of DALYs, indicating that the years of life loss due to PICH were mainly due to premature mortality, which was related to the high PICH mortality rate. Among both men and women and across the age groups, the YLLs were higher than the YLDs in the composition of DALYs, a finding consistent with the composition of DALYs in stroke [23–25]. However, the composition of the DALY rates of PICH patients significantly differed from that of total stroke patients. This study shows that among both men and women and across age groups, the rates of YLLs were higher than the rates of YLDs. In contrast, among stroke patients younger than 80 years, the rate of YLLs was lower than the rate of YLDs because the PICH mortality rate was higher than that of stroke [26, 27].

Reducing PICH cases is the best approach to reducing the PICH disease burden. Our study found that the incidence of PICH in the elderly group decreased, which means that in addition to age, other PICH risk factors are the sum of different decades and exposure experience. Previous studies have shown that hypertension, smoking, drinking, diabetes, increased serum total cholesterol, and high body mass index (BMI) are risk factors for PICH [28]. Implementing measures targeting these primary causes to prevent PICH is an important means to reduce the PICH disease burden. These related risk factors can be intervened manually, not only for the risk factors themselves, but also for reducing or delaying their complications (such as hypertension, diabetes and multiple organ damage), and for reducing or delaying the onset of PICH. Niu Xiaolan et al. [29] analyzed the changes in the risk factors of stroke in the community population in Beijing from 2011 to 2017, and found that while the proportion of people at high risk of stroke was decreasing year by year, the awareness rate, the control rate and the informed person control rate of hypertension, diabetes and dyslipidemia were significantly improved. Tobacco control (via increased taxes, reduced advertising, and banning of smoking in public places), strategies for salt reduction, and evidence-based multidrug strategies to treat those at high risk of cardiovascular disease, could be used to reduce the burden of PICH [30]. Furthermore, it has been studied that the fastest-growing risk factor for stroke between 1990 and 2019 was high body mass index[31]. The prevalence rates of overweight and obesity among young people (26–45 years old) were 35.0% and 12.1%, respectively in Changsha in 2011 [32], which was higher than the national average level among age 18–45 years old people (22.6%, 6.4%, respectively) in 2005 [33]. Thus, weight management for young people may have better significance for reducing the disease burden of PICH. The proportion of DALYs due to hemorrhagic stroke in high-income countries is lower than that in low-income countries because of enhanced public awareness and behaviors related to preventive measures in economically developed regions [34, 35]. Taken together, the measures, including weight control, reasonable diet, and increased physical activity, contribute to reduce the burden of PICH for young people. In addition, according to our results, the incidence and burden of PICH have begun to rise after 50 years old; therefore, controlling blood pressure, blood lipids, and blood sugar stable is an important measure for middle-aged people.

### Limitations of this Study

PICH is characterized by a rapid onset and high mortality. Some patients may die before they have a chance to visit a doctor or arrive at a hospital; in such cases, these data were missed in the outpatient and inpatient records. As a result, the incidence of PICH identified in this study may be lower than the actual incidence in Hunan Province in 2018. Nevertheless, the results of this study indicate that PICH is prevalent and severe in Hunan Province because of the green healthcare initiative for stroke in China, where a stroke center was established in virtually all prefecture-level cities, and better healthcare coverage, which encourages most patients to seek medical attention after the initial onset.

Furthermore, the mortality data in each region lag by 1–2 years. In 2020, mortality data from 2018 were unavailable due to the COVID-19 pandemic. We were unable to analyze the DALYs in each region at this point. Moreover, we focused on PICH and did not retrieve all ICH cases, preventing us from analyzing the proportion of DALYs due to PICH versus all DALYs due to ICH in 2018. Therefore, we used the relevant data from 2017 for the estimations; such estimates serve as a valuable reference for the disease burden of PICH in Hunan Province and resource allocation and optimization for stroke.

## Conclusion

In this study, we estimated the DALYs due to PICH in Hunan Province in 2018. Our results may provide relevant data for the development of policies and measures for the management of PICH disease burden.

## Supplementary Information

Below is the link to the electronic supplementary material.

Supplementary file1 (PDF 65 KB)

## Data Availability

The datasets generated during and/or analyzed during the current study are not publicly available due but are available from the corresponding author on reasonable request.

## Abbreviations

PICH: Primary intracerebral hemorrhage
ICH: Intracerebral hemorrhage
DALYs: Disability-adjusted life years
YLLs: Years of life lost
YLDs: Years lived with disability
GBD: Global Burden of Disease
ICD: Diagnosis code
CT: Computed tomography
MRI: Magnetic resonance imaging
CIs: Confidence intervals
WHO: World Health Organization
BMI: Body mass index
