# Supplementary material for: The Disease Burden of Primary Intracerebral Hemorrhage in Hunan Province, China in 2018
Source: J Epidemiol Glob Health. 2022 May 28;12(3):267–73. doi: 10.1007/s44197-022-00045-5 (PMC9146816; doi:10.1007/s44197-022-00045-5)
Supplement: Supplementary file 1 — Supplementary file1 (PDF 65 KB) [file 44197_2022_45_MOESM1_ESM.pdf]

Supplemental Table 1. YLLs due to PICH (patient-years, 95% CI) and the rate of YLLs (patient-years per 1000 individuals, 95% CI) in Hunan Province in 2018

Age (years)	Men		Women		Total	
	YLLs	Rate of YLLs	YLLs	Rate of YLLs	YLLs	Rate of YLLs
0~	306.7(195.1-418.3)	0.1(0.1-0.2)	193.3(104-282.5)	0.1(0.0-0.1)	500(299.1-700.9)	0.1(0.1-0.2)
5~	3254(2716.2-3791.7)	0.6(0.5-0.8)	1898.5(1482.3-2314.7)	0.4(0.3-0.5)	5152.4(4198.5-6106.4)	0.5(0.4-0.6)
15~	15695.9(14155-17236.7)	2.3(2-2.5)	8086.6(6962.9-9210.4)	1.3(1.1-1.5)	23782.5(21117.9-26447.1)	1.8(1.6-2)
30~	56163.8(53456.4-58871)	6.4(6.1-6.7)	21448.4(19767-23129.8)	2.6(2.4-2.8)	77612.2(73223.4-82000.8)	4.6(4.3-4.8)
45~	112054.4(108499.5-115609.4)	12.7(12.3-13.1)	60812(58129.7-63494.3)	7.2(6.9-7.6)	172866.5(166629.2-179103.7)	10(9.7-10.4)
60~	65745(63929.4-67560.6)	17.3(16.8-17.8)	40006.3(38508.6-41503.9)	11.0(10.6-11.4)	105751.3(102438-109064.5)	14.2(13.8-14.6)
70~	31320.4(30332.6-32308.3)	17.1(16.6-17.7)	21129.8(20251.7-22007.9)	11.6 (11.1-12.1)	52450.2(50584.3-54316.1)	14.4(13.9-14.9)
≥80	7408.6(7063.2-7754)	9.2(8.8-9.6)	6043.7(5700.7-6386.7)	6.1(5.7-6.4)	13452.3 (12763.9-14140.8)	7.5(7.1-7.8)
Total	291948.8(280347.2-303550.1)	7.6(7.3-7.9)	159618.6 (150906.8-168330.2)	4.4(4.2-4.7)	451567.3(431254-471880.3)	6.1(5.8-6.3)

Supplemental Table 1 (continued). YLD due to PICH (person-years, 95% CI) and the rate of YLD (person-years per 1000 individuals, 95% CI) in Hunan Province in 2018

Age (years)	Men		Women		Total	
	YLDs	Rate of YLDs	YLDs	Rate of YLDs	YLDs	Rate of YLDs
0~	12.7(8.1-17.4)	<0.1(0.0-0.0)	8.0(4.3-11.7)	<0.1(0.0-0.0)	20.7(12.4-29)	<0.1(0.0-0.0)
5~	133.9(113.9-153.9)	<0.1(0.0-0.0)	77.3(62-92.7)	<0.1(0.0-0.0)	211.2(175.9-246.5)	<0.1(0.0-0.0)
15~	556(502.8-609.2)	0.1 (0.1-0.1)	275.5(238-312.9)	<0.1(0.0-0.0)	831.4(740.8-922.1)	0.1 (0.1-0.1)
30~	2440.1 (2330.5-2549.7)	0.3(0.3-0.3)	909.7(843.9-975.4)	0.1 (0.1-0.1)	3349.8(3174.5-3525.1)	0.2(0.2-0.2)
45~	5649.8(5475.7-5824)	0.6(0.6-0.7)	2972.3(2846.7-3097.9)	0.4(0.3-0.4)	8622.1(8322.3-8921.9)	0.5(0.5-0.5)
60~	4534.5(4409.8-4659.2)	1.2(1.2-1.2)	2487.3(2394.5-2580.1)	0.7(0.7-0.7)	7021.8 (6804.3-7239.3)	0.9(0.9-1.0)
70~	3127.2(3026.1-3228.4)	1.7(1.7-1.8)	1861.3(1782.5-1940.1)	1.0(1.0-1.1)	4988.5(4808.5-5168.5)	1.4(1.3-1.4)
≥80	1083.3(1027-1139.7)	1.3(1.3-1.4)	779.3(731.2-827.4)	0.8(0.7-0.8)	1862.6(1758.2-1967.1)	1.0(1.0-1.1)
Total	17537.6(16893.8-18181.5)	0.5(0.4-0.5)	9370.5(8903-9838.1)	0.3(0.2-0.3)	26908.2(25796.8-28019.6)	0.4(0.3-0.4)
